# Design and effects of outcome-based payment models in healthcare: a systematic review

**DOI:** 10.1007/s10198-018-0989-8

**Published:** 2018-07-05

**Authors:** F. P. Vlaanderen, M. A. Tanke, B. R. Bloem, M. J. Faber, F. Eijkenaar, F. T. Schut, P. P. T. Jeurissen

**Affiliations:** 1 Radboudumc, Geert Grooteplein Zuid 10, 6525 GA Nijmegen, The Netherlands; 2 Scientific Institute for Quality of Healthcare (IQ Healthcare), Celsus Academy for Sustainable Healthcare, Radboudumc, Nijmegen, The Netherlands; 3 Department of Neurology, Radboudumc, Nijmegen, The Netherlands; 4 Scientific Institute for Quality of Healthcare (IQ Healthcare), Radboudumc, Nijmegen, The Netherlands; 5 Erasmus School of Health Policy and Management, Erasmus University, Rotterdam, The Netherlands

**Keywords:** Outcome-based payment models, Health reform, Payment models in healthcare, Health outcomes, Healthcare costs, Quality of care, I180

## Abstract

**Introduction:**

Outcome-based payment models (OBPMs) might solve the shortcomings of fee-for-service or diagnostic-related group (DRG) models using financial incentives based on outcome indicators of the provided care. This review provides an analysis of the characteristics and effectiveness of OBPMs, to determine which models lead to favourable effects.

**Methods:**

We first developed a definition for OBPMs. Next, we searched four data sources to identify the models: (1) scientific literature databases; (2) websites of relevant governmental and scientific agencies; (3) the reference lists of included articles; (4) experts in the field. We only selected studies that examined the impact of the payment model on quality and/or costs. A narrative evidence synthesis was used to link specific design features to effects on quality of care or healthcare costs.

**Results:**

We included 88 articles, describing 12 OBPMs. We identified two groups of models based on differences in design features: narrow OBPMs (financial incentives based on quality indicators) and broad OBPMs (combination of global budgets, risk sharing, and financial incentives based on quality indicators). Most (5 out of 9) of the narrow OBPMs showed positive effects on quality; the others had mixed (2) or negative (2) effects. The effects of narrow OBPMs on healthcare utilization or costs, however, were unfavourable (3) or unknown (6). All broad OBPMs (3) showed positive effects on quality of care, while reducing healthcare cost growth.

**Discussion:**

Although strong empirical evidence on the effects of OBPMs on healthcare quality, utilization, and costs is limited, our findings suggest that broad OBPMs may be preferred over narrow OBPMs.

**Electronic supplementary material:**

The online version of this article (10.1007/s10198-018-0989-8) contains supplementary material, which is available to authorized users.

## Introduction

In most developed countries, policy makers are searching for payment systems which stimulate the quality of care and reduce healthcare costs. The predominant fee-for-service and diagnosis-related group (DRG) models incentivize volume, and are, therefore, widely considered to be an important reason for rising costs in healthcare [1]. While incentivizing volume can lead to reduced waiting times and better access to healthcare, fee-for-service and DRG models lack incentives for improving quality: providers are paid for the quantity of care that they deliver, not for the impact on the health status of their patients [2]. Since the start of this century, pay-for-performance (P4P) models became popular as a response. In P4P models, reimbursement of healthcare providers explicitly depends on meeting predefined quality targets, which, to date, have largely been based on process and structure indicators [3]. Though models based on these indicators have been studied extensively, evidence that these P4P models are (cost-)effective is limited [4, 5]. In addition, it is still unclear whether the results of initially effective P4P models are sustainable [4–6]. Many authors emphasize the important influence of adequate design features, including the selection of incentivised indicators, on the effectiveness of P4P models [4, 7–15].

Over the last decade, the different shortcomings of P4P models based on structure and process indicators have been addressed by an increased incorporation of outcome indicators. The question is if this increased focus on outcomes has resulted in better quality of care and/or reduced cost growth, or if there are other design features that are (more) important.

However, a comparative evaluation of payment models with an increased focus on outcomes is lacking. Therefore, we conducted a systematic review of the literature on the effects of these new models. Our objective is to synthesize the evidence of the effects on quality of care, healthcare utilization, and healthcare costs. This will lead to better understanding of the consequences of these models, and will help to determine which design features lead to favourable effects, and why. In addition, it might lead to further development and implementation of effective payment models.

In this paper, we use the term ‘outcome-based payment models’ (OBPMs) to denote payment models with a substantial reliance on outcome indicators. Although this term is frequently used in the literature, there is no uniform definition [16, 17]. For example, there is no standard about the minimum use of outcome indicators, while only a few models use outcome indicators exclusively. When creating a definition for OBPMs, we noted that, in P4P models, outcome indicators typically contribute less than 10% to the performance-related incentive payments (see the examples in [9, 16–18]). Based on this finding and on expert opinions in the field (Appendix A3), we choose for a pragmatic approach to consider programmes OBPMs if at least 10% of the performance-related incentive payment is determined by scores on outcome indicators. We adopted the following definition:

An outcome-based payment model is a payment model in healthcare in which the performance-related incentive payments for the healthcare providers depend for at least 10% on outcomes of the provided care, and which is designed to stimulate favourable effects in terms of quality of care or healthcare costs.

We address the following questions: (1) What are the design features of OBPMs and to what extent do they differ from each other? (2) What are the effects of OBPMs on quality of care, healthcare utilization, and healthcare costs?

## Methods

### Inclusion and exclusion criteria

Included articles had to describe the effects on quality of care, healthcare utilization, or healthcare costs of at least one OBPM that matched the definition mentioned in the introduction. In this article, quality of care is assessed by the scores on quality indicators according to the Donabedian framework (structure, process and outcome indicators) [19]. ‘Outcome’ is defined as ‘the effects of care on the health status of patients and populations’ [19]. We do not distinguish between intermediate outcomes (e.g., blood pressure values), final outcomes (e.g., mortality, complication rates, and hospital readmissions), and patient-reported outcomes. ‘Healthcare costs’ are defined according to the definition of the OECD: ‘the sum of expenditure on activities that—through application of medical, pharmaceutical, and nursing knowledge and technology—have certain healthcare-related objectives’ [20].

Articles written in English and published between January 2000 and October 2016 were included. We only included effects that were achieved in OECD countries [21], since the aims and contexts of programmes in other countries are too different to allow a useful comparison. To be as comprehensive as possible, we did not focus on a specific healthcare sector (e.g., in- or outpatient care), despite typical differences in incentive structures that might exist across sectors. There was also no restriction in study design; qualitative studies, quantitative studies, and reviews were all eligible for inclusion. However, articles describing only simulated or expected effects were excluded. Because we expected that many evaluations of OBPMs are not published in scientific peer-reviewed journals, we included governmental and other research reports (provided that they matched our inclusion and exclusion criteria) to ensure a complete inclusion of information. Letters, editorials, and viewpoints that did not contain primary research were excluded.

### Search strategy

We used four data sources to ensure a comprehensive search. First, we searched three databases with scientific literature (Medline, the Cochrane Library, and EMBASE), using the keywords listed in Appendix A1. Second, we consulted websites of relevant governmental and/or scientific agencies (see Appendix A2). Third, we searched through the references of the yielded documents. Finally, we consulted several experts in the field, all of whom responded (see Appendix A3).

### Selection procedure

Titles and abstracts of the documents yielded by the three scientific databases were checked for duplicates and remaining articles were screened for relevance. Full texts of seemingly relevant articles were subjected to the inclusion and exclusion criteria. To determine if a model matched our definition of an OBPM, we sometimes searched for additional information about the model on the Internet via Google, using programme-specific keywords. The selection procedure was done independently by two reviewers. Meetings were held to minimise interobserver bias. Differences were resolved in a discussion between the reviewers, if necessary after consultation of a third reviewer.

Next, articles found on websites of the consulted agencies, articles that were brought to our attention by the consulted experts, and articles retrieved from references of included documents were subjected to the inclusion and exclusion criteria.

### Data extraction

To extract and summarize the data, we developed an extraction form (Appendix B). This form contained the three elements:

- name, country, and period in which the model was operating;
- design features of the payment model;
- effects on quality of care, healthcare utilization and healthcare costs.

A methodological challenge was the fact that payment models tend to change over time, sometimes on an annual basis, e.g., indicators were added or removed, payment structure changed. To address this, we searched for additional information about the changes in programme design over time. If, due to these changes, the model did not meet our definition of OBPM in a specific year, the results achieved in that year were not taken into account. The process of data extraction was performed by two independent reviewers.

### Study appraisal

To appraise the methodological quality of the included quantitative studies, we used the generic and widely applied method described by Downs and Black [22]. In the Downs and Black method, articles receive points on 27 items covering 4 domains: reporting, external validity, internal validity, and power. The more points an article receives, the higher the methodological quality of the article. The maximum number of points is 32 [22]. We chose this generic appraisal method because of the expected heterogeneity of the included study designs, e.g., interrupted time series, observational cohort studies, and cross-sectional studies. To determine the methodological quality of included qualitative studies and reviews, we used the Critical Appraisal Skills Programme checklists [23, 24]. These appraisal methods have been used in other systematic reviews of the effects of payment models in healthcare [4, 25, 26].

The study appraisal was performed by one reviewer; a second reviewer then did an independent review of all qualitative studies and reviews, plus a random selection of 10% of the included quantitative studies. Meetings were held to minimise interobserver bias. Differences were resolved in a discussion between the reviewers, if necessary after consultation of a third reviewer.

## Results

### Included studies

**Fig. 1:**
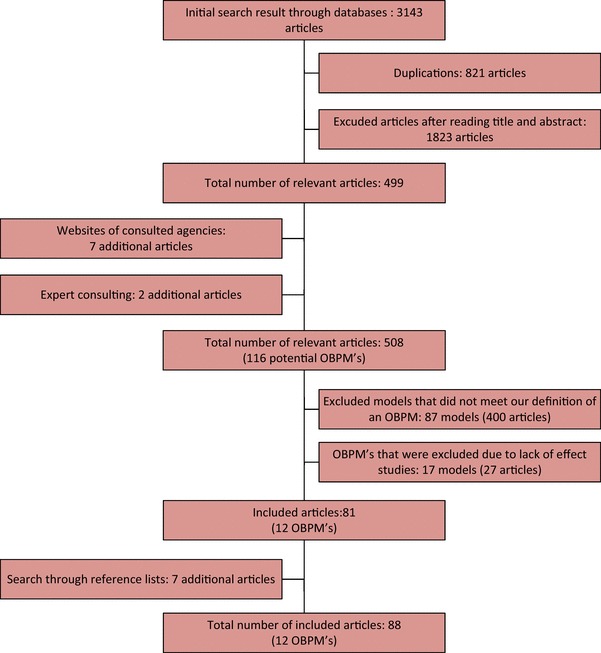
Search flow and results

Figure 1 summarizes the search flow. The 88 included articles contained 75 quantitative studies, 8 qualitative studies, 3 research reports, and 2 reviews. All quantitative studies had a quasi-experimental design (difference-in-difference and case-control design). They had an average Downs and Black score of 11.7 (out of 32) and a standard deviation of 1.9 (Appendix A4). Most points were lost on items about internal validity and statistical power.

One quantitative study contained results for two OBPMs, and one policy report contained results of three OBPMs. The rest of the yielded documents described only one model. In total, we identified 29 OBPMs (Appendix A5), of which 12 could be included for our analysis. Tables 1, 2 provide the general characteristics and the design features of the 12 included OBPMs.

**Table 1:** Characteristics of the 12 included outcome-based payment models

Name, country, period, and references	Healthcare purchaser	Targeted care	Targeted healthcare providers	Outcome indicators and their contribution (in %) to the performance-related payment size
Alternative Quality Contract (AQC), USA, since 2009 [9, 16, 27, 28]	Blue Cross Blue Shield (BCBS) Private; HMO	All care for BCBS insured	Integrated care model: all providers involved in targeted care	Cholesterol levels; HbA1c levels; blood pressure (35.3%)^a^
Commissioning for Quality and Innovation (CQUIN), UK, since 2010 [9, 29]	National Health Service (NHS) Public; single purchaser	Acute care, ambulance service, mental health care, and home care for NHS	Multiple provider model: all providers involved in targeted care	Unknown: differs locally (usually > 10%)
Hospital Quality Incentive Demonstration (HQID), USA, 2003–2009 [9, 16, 30, 31]	Centers for Medicare and Medicaid Services (CMS) Public	Hospital care for Medicare insured (= USA citizens of 65+ age) in 5 clinical areas: heart failure, pneumonia, hip/knee replacements, CABG, acute myocardial infarction	Single provider model: hospitals	30-day mortality; readmission rate; post-ok haemorrhage; post-ok physiologic/metabolic derangement (16.4%)^a^
Hospital Readmission Reduction Program (HRRP), USA, since 2012 [32, 33]	Centres for Medicare and Medicaid Services (CMS) Public	Hospital care for Medicare patients with acute myocardial infarction, heart failure and pneumonia	Single provider model: hospitals	30-day hospital readmissions for acute myocardial infarction, heart failure, pneumonia, and hospital-acquired conditions (100%)
Hudson Health Plan, USA; since 2004 [34]	Hudson Health Plan Private; non-profit	Primary care for diabetes patients enrolled in Hudson Health Plan	Single provider model: primary care physicians	Hba1C levels; blood pressure; cholesterol levels; microalbumin levels (46.7%)
Maryland Hospital-Acquired Condition Program (Maryland HACP), USA, since 2009 [35]	State of Maryland Public	Hospital care of all patients with hospital-acquired conditions (HACs)	Single provider model: hospitals	Hospital-acquired conditions (100%)
Medicare Shared Savings Program (MSSP), USA, since 2012 [9, 36]	Centres for Medicare and Medicaid Services (CMS) Public	All care for patients assigned to participating healthcare organisations	Integrated care model: all participating providers involved in targeted care	Blood pressure; HbA1C levels; cholesterol levels (18.2%)
Palo Alto Medical Clinic P4P Program (PAMC P4P), USA; since 2007 [37, 38]	Palo Alto Medical Foundation (PAMF) Private; non-profit	Primary care of all patients who visit targeted providers	Single provider model: primary care physicians	Blood pressure; HbA1C levels; cholesterol levels (20.0%)
Pioneer Accountable Care Organizations (Pioneer ACO), USA, since 2012 [9, 36]	Centres for Medicare and Medicaid Services (CMS) Public	All care for all patients assigned to participating healthcare organisations	Integrated care model: all participating providers involved in targeted care	Blood pressure; HbA1C levels; cholesterol levels (18.2%)
Quality and Outcomes Framework (QOF), UK, since 2004 [9, 39–43]	National Health Service (NHS) Public, single purchaser	All primary care for NHS insured (= all UK citizens)	Single provider model: primary care physicians	Blood pressure, HbA1C levels; cholesterol levels; lithium levels (20.8%)
Value-Based Purchasing (VBP), USA, since 2012 [9, 44]	Centres for Medicare and Medicaid Services (CMS) Public	Hospital care for CMS insured (= USA low income citizens or 65+ age)	Single provider model: hospitals	30-day mortality, catheter associated urinary tract infections, central line-associated blood stream infections, surgical site infections, MRSA or C. Difficile infections and elective deliveries (2013: 0%; 2014: 25%; 2015: 30%; 2016: 50%; 2017: 50%)
Value Incentive Program (VIP), Korea; since 2007 [16, 45, 46]	National Health Insurance of Korea (NHIK) Public, single purchaser	Hospital care of NHIK insured (= all Korean citizens) in 3 clinical areas: Acute Myocardial Infarction (AMI), Caesar Sections, and acute stroke (since 2012)	Single provider model: hospitals	30-day mortality (30%, AMI only)

^a^These percentages are averages, since this model uses separate indicator sets for different care settings

**Table 2:** Design features of identified outcome-based payment models

	Indicators				Measurement				Payments					Refs.
	Type of indicators used	No. of indicators (of which outcome indicators)	Extra weight to outcome indicators	Net contribution of outcome indicators to quality score	Scores reported by	Risk-mitigating measures	Publication of scores	Feedback to providers	Incentive types	Requirements for bonus	Requirements for penalty	Requirements for shared savings	Maximum bonus/penalty size	
Narrow OBPMs														
CQUIN	S, P, O	Differs locally	Differs locally	Differs locally (29% average)	Providers	Risk-adjustment per indicator	?	?	P	n.a.	Differs locally	n.a.	− 0.5% (2009) to − 2.5% (2012) of contract income	[9, 47]
HQID	S, P, O	AMI: 9 (1) CABG: 8 (3) HF: 4 (0) Pneu: 7 (0) H&K: 6 (3)	No	AMI: 11.1% CABG: 37.5% HF: 0% Pneu: 0% H&K: 50.0%	Providers	Case mix + exception reporting	Yes	Yes, annual	B, P	Top 20% overall; top 20% improvement	Bottom 20%	n.a.	B: + 2% on DRG P: − 2% on DRG	[9, 16, 30, 31]
HRRP	O	3(3)	n.a.	100%	?	Adjusted for age, sex, and co-morbidities	?	?	P	n.a.	Below 3-year average readmission rate	n.a.	2012–2014: max − 1% p/DRG 2015+: max − 3% p/DRG	[32, 33]
Hudson Health Plan	P, O	Diab: 14 (4)	$140/$300 per patient	46.7%	Providers	?	No	Yes, annual	B	None: fixed price per indicator per patient	n.a.	n.a.	$300,– per patient	[34]
Maryland HACP	O	HACs: 49 (49)	No	100%	Providers	Corrected for nr of HACs in Y-1	?	?	B, P	?	?	n.a.	B: ? P: − 2% of total revenue	[35]
PAMC P4P	S, P, O	15 (3)	No	20.0%	Health records	Case mix	Yes	Yes, quarterly	B	Achieving minimal target per indicator	n.a.	n.a.	$5000,– per year	[37, 38]
QOF	S, P, O	’04: 146 (10) ’06: 135 (?) ’14: 81 (17)	Yes	’14: 20.8%	Providers	Exception reporting	Yes	Yes, annual	B	Achieving minimal target per indicator	n.a.	n.a.	+ 25% of budget (after 2014: + 17%)	[9, 39–43]
VBP	S, P, O	2013: 12 (0) 2014–2015: 15 (3) 2016+: 17 (5)	No	2013: 0% 2014: 25% 2015: 30% 2016: 50% 2017: 50%	Providers	Corrected for age, sex, CD	Yes	Yes	B, P	None: general + 1% (2013)/+ 2% (2017) per DRG	None: general − 1% (2013)/− 2% (2017) per DRG	n.a.	B: + 1% (2013)/2% (2017) P: − 1% (2013)/− 2% (2017) per DRG	[9, 44]
VIP	P, O	AMI: 6 (1) CS: 1 (0) Stroke: 11 (0)	AMI: 1.8×	AMI: 30% CS: 0% Stroke: 0%	Claims data	Corrected for age	Yes	Yes, annual	B, P	Top 20% overall; top 20% improvement	Below threshold (= below 80% best score in Y-2)	n.a.	First phase: B: +1% on DRG P: − 1% on DRG Second phase B: + 1% on DRG P: − 1% on DRG	[16, 45, 46]
Broad OBPMs														
AQC	S, P, O	pc: 32 (5) sc: 33 (5)	pc: 3× sc: 3×	pc: 35.7% sc: 34.9%	Providers	Corrected for age, CD	?	Yes, monthly	B, SS	> Median score; s-shaped relation	n.a.	None	B: + 10% of global budget for highest target SS: no max	[9, 16, 27, 28]
MSSP	P, O	33 (6)	No	18.2%	Patients, providers	No downside risk (option); population correction	?	?	SS	n.a.	n.a.	Target on quality indicators	B: 60% of savings (50% if no downside risk) to 7.5% Medicare spending P: 10% of loss	[9, 36]
Pioneer ACO	P, O	33 (6)	No	18.2%	Patients, providers	Lim downside risk; popul. correction	?	?	B, SS	?	n.a.	Target on quality indicators	B: ? P: ?	[9, 36]

*n.a*. not applicable, *?* unknown, *O* outcomes, *P* process, *S* structure, *AMI* acute myocardial infarction, *CABG* coronary artery bypass graft, *CS* Caesar section, *Diab* diabetes, *H&K* hip and knee replacement, *HACs* hospital-acquired conditions, *HF* heart failure, *pc* primary care, *Pneu* pneumonia, *sc* secondary care, *AMI* acute myocardial infarction, *pc* primary care, *sc* secondary care, *CD* chronic diseases, *lim* limited, *B* bonus, *P* penalty, *SS* shared savings

Based on the general characteristics (Table 1) and the design features (Table 2), we identified two types of OBPMs. We called the first-group ‘narrow OBPMs’. The models comprising this group focus exclusively on explicit financial incentives for objectively measured quality, with the incorporation of relatively many outcome indicators (i.e., pertaining to > 10% of performance-related reimbursement). In these models, providers earn bonuses and/or suffer penalties based on their scores on a predefined set of indicators. These models typically target one provider type (e.g., hospitals and primary care physicians) and/or specific clinical areas (e.g., care for acute myocardial infarction). The other group of models is called ‘broad OBPMs’. These models encompass the entire provider payment by combining global budgets and shared savings incentives with explicit financial incentives for quality indicator scores. This group of models generally targets multidisciplinary provider groups providing different types of care for their patient population.

### Effects of OBPMs

Most articles (58) describe effects on quality of care only, 9 articles on healthcare utilization or healthcare costs, and 21 articles on both quality and utilization/costs. The follow-up period varies from 9 months to 7 years. Table 3 summarizes the effects of OBPMs on quality of care and healthcare utilization/costs.

**Table 3:** Effects of OBPMs on quality of care and healthcare utilization/costs

Model	Quality of care	Healthcare utilization/costs	Number of studies	Downs and Black score: mean (SD)
Narrow OBPMs				
CQUIN	+	?	3	9.0 (1.0)
HQID	Mixed^a^	−	13	11.4 (1.6)
HRRP	+	?	2	9.0 (1.0)
Hudson Health Plan	Mixed	−	2	13.0 (0)
Maryland HACP	+	?	1	10.0 (0)
PAMC P4P	−	?	2	10.5 (2.5)
QOF	+	−^b^	43	11.9 (1.9)
VBP	−	?	9	11.5 (2.3)
VIP	+	?	3	12.0 (2.0)
Broad OBPMs				
AQC	+	+	10	12.4 (1.1)
MSSP	+	+	2	11.0 (0)
Pioneer ACO	+	+	2	11.0 (0)

Effects are regarded positive when at least 65% of the articles find that a significant improvement in quality of care or reduced healthcare costs. When the majority of studies found that the quality of care did not improve (or worsened) or healthcare costs increased, we considered the effect negative

*?* unknown

^a^After 3 years, the HQID adopted some design changes. In the first-phase quality of care improved, the second phase was less successful

^b^One of the aims of this programme was to increase the income of general practitioners substantially

### Effects on quality of care

Regarding the effects of the models on quality of care, evidence is available for all 12 models. Of the 88 included studies, 79 targeted quality of care.

#### Incentivised indicators

All three broad OBPMs showed improvements on the incentivised indicators. Process indicators improved in multiple studies [9, 27, 28, 48–50], while improvement of outcome indicators was only found for diabetes and vascular care in one study (AQC) [49]. No improvement was found in outcome indicators for substance use disorder patients [51], emergency department use (both AQC) [52], or hospital readmissions (Pioneer ACO) [53].

For the narrow OBPMs, five out of nine models showed positive results on the incentivised indicators (CQUIN, HRRP, Maryland HACP, QOF, and VIP) [33, 35, 40, 42, 45, 54–59]; one showed mixed results (Hudson health plan) [34, 60]; in two models, no significant effect was found (PAMC, VBP) [38, 61–64]. In the remaining model (HQID), some improvements were observed in the first phase of the programme (first 3 years), but, after some alterations in the design, these improvements did not last [9, 31, 65–68].

As, in the broad OBPMs, process indicators showed larger improvements than outcome indicators. Five out of nine programmes (CQUIN, HQID, Hudson Health plan, QOF, and VIP) reported improvements in certain process indicators [9, 40, 42, 45, 54–60, 65, 66, 69–72], while four (HRRP, Maryland HACP, QOF, and VIP) showed improvements in outcomes [33, 35, 56, 59, 71–75]. Two of these could not show improvements in process indicators, because these models only included outcome indicators (HRRP and Maryland HACP). Outcome indicators that showed improvements were hospital readmissions after acute myocardial infarction (HRRP) [33], hospital-acquired conditions (Maryland HACP) [35], blood pressure and lab results for diabetes and renal disease (both QOF) [56, 71–73], mortality after stroke (VIP) [59], emergency hospital admissions (QOF) [74], and homecare placements for patients with dementia (QOF) [75]. However, most outcome indicators did not significantly improve [34, 62, 63, 76, 77], the mortality rate in particular remaining unaffected (in HQID, QOF, and VBP) [31, 62, 63, 65, 67, 68, 78].

While the effects of broad OBPMs on quality of care increased over time [9, 27, 28, 48], positive effects of narrow OBPMs tended to be short-lived. In two broad OBPMs (AQC and Pioneer ACO), effects on the incentivised indicators increased over the years [9, 27, 28, 48]. In contrast, two narrow OBPMs (HQID and QOF) showed ceiling effects. For HQID, this occurred after a significant revision of the incentive structure [9, 66, 79, 80], while for QOF diabetes and asthma indicators already reached a ceiling after the first year [69]. For most of the other indicators in the QOF, ceiling effects emerged after years 2 or 3 [42, 70], when many GP practices exceeded the quality thresholds for maximum incentive payments [55]. However, the percentage of hospital emergency admissions continued to decrease as a result of the QOF [74].

#### Relevant provider and patient characteristics

Private providers and providers with low baseline quality scores improved their performance the most (Hudson Health plan, MSSP, Pioneer ACO, QOF, VBP, VIP) [45, 59, 60, 71, 81–84], although some studies concerning the VBP report relatively poor performance of initially low-scoring providers, and in HQID safety net hospitals performed relatively poorly [9, 61–63, 67, 79, 80]. Among the narrow OBPMs, three models (HQID, Hudson Health plan, QOF) show that large providers outperform smaller ones [30, 60, 85]. In the VBP model, this scale effect is mixed [83, 84, 86].

It remains unclear if high-need patients benefit more from OBPMs than other patients. In the AQC, children with special needs benefitted more than others from preventive paediatric care [50]. In the QOF, quality of care for diabetics with co-morbidities improved more than for those without co-morbidities [87]. In contrast, mental health centres (AQC), nursing homes (QOF), and hospitals with more Medicare and Medicaid patients (VBP) showed significantly lower quality scores after introduction of a OBPM [49, 51, 84, 88]. In the Hudson Health Plan, there was no change in quality of care for patients both with and without co-morbidities [34].

### Effects on healthcare utilization and costs

Regarding the effects on healthcare utilization and healthcare costs, three (out of nine) narrow OBPMs are included (13 studies) in the analysis. Of the broad OBPMs, all three models were included (17 studies).

#### Healthcare utilization

For five models (AQC, HQID, Hudson Health Plan, Pioneer ACO, and QOF), data were available about effects on healthcare utilization. Two out of three narrow OBPMs showed an increase in healthcare utilization. Prescription of preventive drugs increased (antibiotics in HQID [65] and antihypertensive drugs in the QOF [89]). Moreover, the number of newly diagnosed diabetics who started with medication increased (QOF) [90]. In the Hudson Health Plan, no significant change in healthcare utilization was found [34].

Contrary to the narrow OBPMs, the two broad OBPMs showed a reduction in healthcare utilization. For the AQC, reductions among Medicare patients were reported in emergency department use, the use of outpatient care, office visits, minor procedures, imaging, and diagnostic tests [91]. This is in line with the reduction of healthcare utilization found 4 years after the introduction of the AQC [48]. However, there was no significant impact on the use of pharmaceuticals [92], while small increases were reported for the use of mental health services [49] and emergency departments [52]. For the Pioneer ACO programme, a reduction in inpatient services was found [53].

#### Healthcare costs

All three broad OBPMs (AQC, MSSP, Pioneer ACO) showed a cost saving based on the incentives of the programme [9, 27, 28, 48, 53, 91]. The MSSP led to a cost saving of about $385 million within 1 year, while the Pioneer ACO reached a comparable cost reduction after 2 years [9, 53]. For the third model (AQC), two out of six studies did not find an effect on healthcare costs [50, 52], while four studies that were performed later found savings of 1.9, 3.3, and 6.8% after 1, 2, and 4 years after introduction, respectively [27, 28, 48, 91].

In broad OBPMs, the cost containment effects increased over time. Several studies reported no or small cost reductions in the first years of the AQC programme [27, 52, 93], while these reductions increased after 1 or 2 years [28, 48, 91]. For the Pioneer ACO programme, one study found similar effects [9], but another study reported the opposite [53]. For the narrow OBPMs, no longitudinal evaluation studies were available with respect to the impact on costs.

Of the narrow OBPMs, costs increased in all three models for which results are available. This is due to the bonus payments [41, 60, 68, 78, 94]. The HQID does not report any significant effect on healthcare costs, but, in the calculation, the $17 million that was spent on bonus payments was not taken into account [68, 94]. Hudson Health Plan, a relatively small programme, spent over $1 million on bonus payments [60]. In the QOF (where a substantial income increase for general practitioners was one of the objectives), over £5 billion was spent in the first 7 years of the programme [41, 78], resulting in a 26–40% increase of income for general practitioners [69, 95].

### Unintended consequences

For four models (AQC, HQID, Maryland HACP, and QOF), studies were available about effects on non-incentivised indicators. For broad OBPMs, data are only available for the AQC. The included studies for this model showed no obvious effect (positive nor negative) on non-incentivised indicators [50, 91]. In contrast, for the narrow OBPMs, some signs of negative effects exist: while HQID shows no effects on not included indicators [65, 79], in the Maryland HACP, the incidence of non-incentivised hospital-acquired conditions increased [35]. In the QOF, there was no change in mortality for either incentivised or non-incentivised diseases [78], but (non-incentivised) continuity of care decreased [69]. Another study regarding the QOF showed an initial improvement in non-incentivised indicators for asthma, diabetes, and vascular diseases, but, after 2 years, these effects decreased to below baseline level [42].

In three narrow models (HQID, Hudson Health plan, QOF), the effects on ethnic and social disparities were analysed, finding little to no improvement, and sometimes a deterioration. In HQID, the existing gap on process quality closed between blacks and whites, but differences in mortality remained [31]. In the Hudson Health Plan, the existing disparities in immunisation rates remained [60]. For QOF, seven out of nine studies found no effects on existing social or ethnic disparities [40, 56, 70, 73, 96–98]. One study showed a decrease between deprived and not deprived patients [41], while another noticed an increasing gap between socioeconomic groups [77].

For the HQID, the HRRP, and the QOF (all narrow OBPMs), several studies examine whether or not providers have been trying to abuse the model by directly or indirectly manipulating the performance scores (gaming). In general, there is a little evidence that this occurred on a large scale. For HQID and HRRP, no evidence was found that hospitals delay readmissions, alter discharge statuses, limit the access for high-risk patients, or focus on the most profitable measures [33, 99, 100]. In the QOF, the generally low levels of exception reporting suggest that large-scale gaming is uncommon [40, 76, 101–104], although some suspect variations in performance scores were noticed [101, 102].

## Discussion

### Summary of principal findings

This review provides an evidence synthesis of the characteristics and effectiveness of 12 OBPMs. Based on differences in design features, two groups of OBPMs were distinguished: narrow OBPMs, which only contain explicit financial incentives for objectively measured quality performance; and broad OBPMs, which combine global budgets and risk sharing for multidisciplinary provider groups with explicit financial incentives for quality. Although only three broad OBPMs could be included in this review, their effects on both quality of care and healthcare utilization/costs are particularly favourable when compared to the narrow OBPMs. In addition, these effects improved over time in the broad OBPMs, while the effects of narrow OBPMs tended to be short-lived. We also found that process indicators showed larger improvements than outcome indicators in both groups of OBPMs. Other findings were: larger private providers and providers with initially poor quality scores tended to score better than other providers; high-need patients did not seem to benefit more from OBPMs than other patients; broad OBPMs had a little effect on non-incentivised indicators, while there are signs that non-incentivised indicators may deteriorate in the narrow OBPMs; narrow OBPMs did not seem to decrease social or ethnic disparities; and narrow OBPMs do not seem to lead to gaming on a large scale.

### Explanations and comparisons to the existing literature

In both groups of OBPMs, process indicators showed larger improvements than outcome indicators. In a way, this may be considered disappointing as it raises the question what the value is of focussing financial incentives on outcomes. One explanation is that outcomes are generally more difficult to influence by providers than processes. Another explanation is that improvements in processes may precede improvements in outcomes, especially in the short term. However, although some studies suggest that the link between processes and outcomes is often not straightforward [105]. Finally, the improvements on indicator scores could be due to ‘signalling power’: the implementation of a payment model can lead to increased attention to the incentivised indicators. This attention, rather than the design features of the payment model, could lead to improvements on easy to influence (process) indicators. Nonetheless, the fact that processes improve is positive, given that many earlier evaluations of P4P programmes (which have focused mainly on processes) show mixed effects on process indicators [4].

The broad OBPMs showed increasing improvements on quality indicators over time, while the effects of the narrow OBPMs tend to be short-lived. This may be due to broad OBPMs generally being less prone to ceiling effects due to a design in which explicit incentives based on objectively measured indicators are combined with more general payment mechanisms (i.e., global budgets with risk-sharing arrangements). In addition, the finding that relatively poor performers improve more is another indication of the existence of ceiling effects, which are reported in some of the included models [9, 42, 55, 66, 69, 70, 79, 80].

We also found that cost savings in broad OBPMs tend to increase over time. In addition, narrow OBPMs typically show increases in healthcare utilization, while broad OBPMs show reductions. These effects might be explained by the additional focus on cost containment in broad OBPMs (i.e., global budgets and risk sharing), while narrow OBPMs focus on quality alone.

Literature on P4P models shows results comparable to our findings on narrow OBPMs: there is evidence that both types of models increase (process) quality of care, although results are mixed and there is no evidence that non-incentivised indicators improve [4]. This might be due to similarities in the design: despite the incorporation of more outcome indicators, the working mechanism of narrow OBPMs is often analogous to that of P4P models (i.e., bonuses or penalties for achieving predefined targets with respect to explicitly measured quality indicators).

We found that larger private providers and providers with initially poor quality scores tend to score better than other providers. A possible explanation is that large private providers and providers with low baseline quality have more improvement potential. Moreover, these findings might be influenced by the ceiling effects found in two models (HQID and QOF). In these models, it was relatively easy to achieve a maximum score on some indicators. The distance to these maximum scores from the baseline (i.e., the achieved improvements) is larger in initially low-scoring providers. On the other hand, providers with relatively many minority patients or with patients with a lower socioeconomic status are known to have poorer quality metrics. Financial incentives run the risk of exacerbating these disparities across providers. For example: there is evidence that safety net hospitals suffer more from the financial penalties introduced by P4P than other hospitals [106].

### Strengths and limitations

This review has multiple strengths and limitations. The strengths are: (1) this is the most comprehensive review on OBPMs to date, comparing 12 different OBPMs from 3 different countries; (2) this review has been conducted systematically and multiple data sources were used; (3) the reviewed studies have a relatively high average level of evidence, since all included quantitative studies adopted a quasi-experimental design. However, as in the previous reviews on payment models [26], experimental studies are lacking. This is largely due to the nature of the intervention (i.e., payment models), which often precludes experimental study designs. In addition, for 8 of the 12 models, only up to 3 studies were available. For these models, the results on quality of care or healthcare costs have a limited scientific base.

The use of our definition of OBPMs results in four limitations. First, the required minimum 10% dependence on outcomes set by the definition is an arbitrary cut-off point; it does not take the total size of the performance-related reimbursement into account. There is also no evidence for a critical cut-off point in incentive size related to effectiveness. Setting the cut-off point at a lower percentage might have resulted in the inclusion of more programmes, possibly in more countries. However, the 10% threshold seems to allow a reasonably effective distinction between more and less outcome-based payment models.

Second, in five of the included programmes (AQC, CQUIN, HQID, VBP, and VIP), we could not determine with absolute certainty if at least 10% of the total incentive payments were always linked to outcome indicators, since these models use separate indicator sets in different geographical regions or care settings. Nevertheless, excluding payment models of which we know that they match our definition in almost all regions or care settings would harm the generalisation of our results. We only included OBPMs when the information at our disposal consistently confirmed that the model matched our definition and that there were no major differences in specific regions or care settings. This was the case for all five aforementioned OBPMs.

Third, we acknowledge that incentives emanating from payments linked to good scores on outcome indicators might be weaker in included OBPMs with small total incentive payment sizes (e.g., the HRRP) than in excluded models with relatively large total incentive payments but in which less than 10% of these payments are linked to outcomes. However, incorporating the size of these payments into the definition of OBPMs is practically impossible and would lead to an unworkable definition, since the required information is often not available, especially in payment models with complex designs.

A final limitation of our review concerns the generalisation of our findings. First, comparing different outcome measures, used in different OBPMs, is not ideal. Some outcome indicators may have more improvement potential than others, and the existence of clear guidelines can increase this potential. Furthermore, some indicators of the HRRP and the VBP programme overlap, since both programmes are implemented in the context of the USA Medicare programme.

Second, our review includes OBPMs from both in- and outpatient sector, which operate differently. Specifically, they are subject to different payment and billing systems, which affect the incentive structure. In addition, OBPMs in the outpatient sector tend to distribute relatively more money than OBPMs in the inpatient sector. Nonetheless, it is useful to use a broader scope by including both sectors.

Third, the effects of the payment models are likely to be influenced by contextual factors. The introduction of OBPMs is often part of a larger policy package, such as increased registration, public reporting, or implementation of feedback systems. Effects can also be influenced by the healthcare system of the involved country. The fact that models are from different countries leads to challenges in drawing conclusions. However, it must be highlighted that 9 out of the 12 models in this review are from the USA. Although this makes a comparison between these nine models easier, the USA is a country with exceptionally high healthcare costs. Positive effects on healthcare costs might, therefore, be easier to achieve than in other countries. Consequently, extrapolation of findings from USA-based studies to other healthcare systems is hard.

## Conclusions

OBPMs are at the centre of the debate on the future of healthcare reimbursement. It is one of the theoretical underpinnings of the movement towards value-based healthcare which seeks for more quality of care and value against the ‘lowest’ possible costs [107]. We conclude that an increased focus on outcome indicators alone is unlikely to result in an increased effectiveness of payment models: other design features also influence the effects on quality of care and healthcare costs. Specifically, our main findings suggest that OBPMs which combine global payments and risk-sharing with explicit bonuses or penalties based on (outcome) indicator scores have most potential to contribute to value. Based on our results, these ‘broad’ OBPMs seem to be more (cost-)effective than the ‘narrow’ OBPMs, as in the latter group evidence of improved quality is less consistent and tends to be short-lived, and evidence for decreases in healthcare costs is lacking. Despite the limitations of our approach and the fact that we still know little about the interaction between costs and quality, we feel that we can recommend broad OBPMs. However, given that we could only include three broad OBPMs, which have all been implemented more recently than the ‘narrow’ OBPMs and all in the USA, more rigorous evaluations of broad OBPMs are required to strengthen this conclusion, preferably in a different context than that of the USA.

## Electronic supplementary material

Below is the link to the electronic supplementary material.

Supplementary material 1 (VSD 69 KB)

Supplementary material 2 (DOCX 50 KB)

Supplementary material 3 (XLSX 91 KB)
