# Supplementary material for: Design and effects of outcome-based payment models in healthcare: a systematic review
Source: Eur J Health Econ. 2018 Jul 5;20(2):217–32. doi: 10.1007/s10198-018-0989-8 (PMC6438941; doi:10.1007/s10198-018-0989-8)
Supplement: Supplementary file 2 — Supplementary material 2 (DOCX 50 KB) [file 10198_2018_989_MOESM2_ESM.docx]

# Appendix A

## Supplementary material

**1. Search string**

*(Outcome*[it] OR quality*[it] OR performance*[it] OR value*[it] OR readmission*[it] OR mortality [it] OR complication*[it])*

*AND*

*(incentiv*[it] OR pay*[it] OR fund[it] OR funding[it] OR funds[it] OR remunerat*[it] OR reimburs*[it] OR financ*[it] OR fee[it] OR fees[it] OR purchas*[it] OR buy*[it] OR contract[it] OR contracts[it] OR contracting[it])*

*AND*

*(model[tiab] OR models[tiab] system[tiab] OR systems[tiab] scheme[tiab] OR schedule*[tiab] OR reform[tiab] OR reforms[tiab] OR program[tiab] OR programme[tiab] OR programs[tiab] OR programmes[tiab]OR framework*[tiab] OR contract[tiab] OR contracts[tiab] OR contracting[tiab] OR project*[tiab])*

**2. Table of consulted agencies**

| *Agency name* | *Country* |
| --- | --- |
| Agency for Healthcare Research and Quality (AHRQ) | USA |
| Australian Institute of Health and Welfare (AIHW) | Australia |
| Bertelsmann Foundation | Germany |
| Commonwealth Fund | USA |
| Health Foundation | UK |
| Institute for Healthcare Improvement (IHI) | USA |
| Institute for research and information in health economics (IRDES) | France |
| King’s Fund | UK |
| Leapfrog Group | USA |
| National institute for Health and Care Excellence, (NICE) | UK |
| National institute for Health and Medical research (INSERM) | France |
| Nuffield Trust | UK |
| Organisation for Economic Co-operation and Development (OECD) | - |
| Robert Bosch Foundation | Germany |
| Robert Wood Johnson Foundation (RWJF) | USA |
| Swedish Institute: College of Health Sciences | Sweden |
| United States Health Information Knowledgebase (USHIK) | USA |
| World Health Organisation (WHO) | - |

**3. List of consulted experts**

| *Consulted experts* |  |
| --- | --- |
| Prof. M. Rosenthal | Professor of Health Economics and Policy and Associate Dean for Diversity at Harvard T.H. Chan School of Public Health, Cambridge, Massachusetts, USA |
| Prof. F.E. Schut | Professor of Health Economics and Health Policy at the Erasmus School of Health Policy and Management (iBMG), Erasmus University of Rotterdam, The Netherlands |
| Dr. F. Eijkenaar | Assistant Professor at the Erasmus School of Health Policy and Management (iBMG), Erasmus University of Rotterdam, The Netherlands |

**4. Quality assessment score**

| Quantitative studies  (Downs & Black score) | Total score | Scores on individual items | | | | |
| --- | --- | --- | --- | --- | --- | --- |
|  |  | **Reporting**  *(max 11)* | **External validity** *(max 3)* | **Internal validity** | | **Power**  *(max 5)* |
|  | *(max 32)* |  |  | **Bias** *(max 7)* | **Confounding** *(max 6)* |  |
| Afendulis 2014 | **14** | 7 | 1 | 4 | 2 | 0 |
| Alshamsan 2012 | **14** | 7 | 0 | 4 | 3 | 0 |
| Barry 2016 | **13** | 7 | 1 | 3 | 2 | 0 |
| Bhattacharyya 2008 | **10** | 5 | 0 | 4 | 1 | 0 |
| Calikoglu 2012 | **10** | 4 | 0 | 3 | 3 | 0 |
| Campbell 2009 | **13** | 6 | 0 | 4 | 3 | 0 |
| Chatfield 2016 | **9** | 4 | 1 | 3 | 1 | 0 |
| Chien 2010 | **13** | 7 | 0 | 3 | 3 | 0 |
| Chien 2012 | **13** | 7 | 0 | 3 | 3 | 0 |
| Chien 2014 | **11** | 5 | 1 | 3 | 2 | 0 |
| Chung 2010a | **13** | 4 | 2 | 4 | 3 | 0 |
| Chung 2010b | **8** | 4 | 0 | 3 | 1 | 0 |
| Crawley 2009 | **10** | 6 | 0 | 3 | 1 | 0 |
| Dalton 2011 | **15** | 7 | 1 | 4 | 3 | 0 |
| Das 2016 | **12** | 6 | 2 | 3 | 1 | 0 |
| Doran 2006 | **14** | 8 | 1 | 4 | 1 | 0 |
| Doran 2008a | **13** | 7 | 1 | 4 | 1 | 0 |
| Doran 2008b | **11** | 6 | 1 | 4 | 0 | 0 |
| Doran 2011 | **15** | 7 | 1 | 4 | 3 | 0 |
| Doran 2012 | **12** | 5 | 2 | 2 | 3 | 0 |
| Epstein 2014a | **14** | 8 | 0 | 3 | 3 | 0 |
| Epstein 2014b | **12** | 7 | 0 | 3 | 2 | 0 |
| Figueroa 2016 | **17** | 9 | 3 | 3 | 2 | 0 |
| Fleetcroft 2012 | **12** | 7 | 0 | 4 | 1 | 0 |
| Gallagher 2014 | **15** | 8 | 2 | 4 | 1 | 0 |
| Gemmell 2009 | **16** | 9 | 1 | 4 | 2 | 0 |
| Gilman 2015 | **11** | 5 | 1 | 3 | 2 | 0 |
| Gravelle 2010 | **13** | 8 | 1 | 3 | 1 | 0 |
| Greene 2015 | **11** | 7 | 0 | 3 | 1 | 0 |
| Guthrie 2006 | **10** | 4 | 1 | 4 | 1 | 0 |
| Harrison 2014 | **12** | 7 | 1 | 3 | 1 | 0 |
| Jha 2012 | **12** | 7 | 0 | 3 | 2 | 0 |
| Kahn 2015 | **10** | 3 | 0 | 4 | 3 | 0 |
| Karunaratne 2013 | **10** | 6 | 0 | 3 | 1 | 0 |
| Kasteridis 2016 | **14** | 8 | 1 | 3 | 2 | 0 |
| Kendrick 2015 | **10** | 6 | 0 | 3 | 1 | 0 |
| Kontopantelis 2012 | **10** | 5 | 0 | 3 | 2 | 0 |
| Kontopantelis 2016 | **10** | 5 | 1 | 3 | 1 | 0 |
| Kristensen 2013 | **10** | 6 | 0 | 2 | 2 | 0 |
| Kruse 2012 | **14** | 7 | 2 | 3 | 2 | 0 |
| Lee 2011 | **9** | 5 | 0 | 3 | 1 | 0 |
| Lee 2012 | **14** | 6 | 1 | 4 | 3 | 0 |
| MacBride-Stewart 2008 | **11** | 6 | 1 | 3 | 1 | 0 |
| McWilliams 2013 | **13** | 8 | 1 | 3 | 1 | 0 |
| Mellor 2016 | **8** | 4 | 0 | 3 | 1 | 0 |
| Millett 2007a | **11** | 7 | 0 | 3 | 1 | 0 |
| Millett 2007b | **12** | 7 | 0 | 3 | 2 | 0 |
| Millett 2008 | **12** | 6 | 0 | 3 | 3 | 0 |
| Millett 2009a | **11** | 6 | 0 | 3 | 2 | 0 |
| Millett 2009b | **8** | 4 | 0 | 3 | 1 | 0 |
| Nattinger 2016 | **11** | 5 | 1 | 3 | 2 | 0 |
| Nicholas 2011 | **11** | 5 | 0 | 4 | 2 | 0 |
| Ramirez 2016 | **11** | 6 | 0 | 3 | 2 | 0 |
| Ryan 2009 | **12** | 5 | 1 | 4 | 2 | 0 |
| Ryan 2012 | **11** | 5 | 1 | 3 | 2 | 0 |
| Ryan 2014 | **10** | 4 | 1 | 3 | 2 | 0 |
| Ryan 2015 | **12** | 7 | 0 | 3 | 2 | 0 |
| Ryan 2016 | **12** | 6 | 1 | 3 | 2 | 0 |
| Serumaga 2011 | **12** | 7 | 1 | 3 | 1 | 0 |
| Shah 2011 | **13** | 5 | 2 | 3 | 3 | 0 |
| Sharp 2013 | **13** | 6 | 1 | 4 | 2 | 0 |
| Shih 2014 | **10** | 6 | 0 | 3 | 1 | 0 |
| Shlebak 2016 | **8** | 5 | 0 | 2 | 1 | 0 |
| Simpson 2011 | **8** | 4 | 0 | 3 | 1 | 0 |
| Song 2011 | **13** | 7 | 1 | 3 | 2 | 0 |
| Song 2012 | **11** | 5 | 1 | 3 | 2 | 0 |
| Song 2014 | **11** | 5 | 1 | 3 | 2 | 0 |
| Strong 2009 | **13** | 5 | 2 | 4 | 2 | 0 |
| Stuart 2016 | **13** | 6 | 1 | 3 | 3 | 0 |
| Vaghela 2009 | **12** | 7 | 1 | 2 | 2 | 0 |
| Wang 2006 | **13** | 6 | 2 | 3 | 2 | 0 |
| Werner 2011 | **9** | 4 | 0 | 4 | 1 | 0 |
| Whalley 2008 | **12** | 6 | 0 | 4 | 2 | 0 |
| Yang 2016 | **10** | 6 | 0 | 3 | 1 | 0 |
| Zhao 2015 | **10** | 5 | 0 | 3 | 2 | 0 |

| Qualitative studies  (CASP method) | scores on individual items | | | | | | | | | |
| --- | --- | --- | --- | --- | --- | --- | --- | --- | --- | --- |
|  | **1** | **2** | **3** | **4** | **5** | **6** | **7** | **8** | **9** | **10** |
|  |  |  |  |  |  |  |  |  |  |  |
| Campbell 2008 | yes | yes | can't tell | can't tell | yes | yes | yes | yes | yes | highly valuable |
| Chien 2016 | no | yes | can't tell | yes | can't tell | yes | yes | can't tell | no | limited |
| Edwards 2007 | yes | yes | yes | yes | yes | can't tell | yes | no | yes | valuable |
| Hannon 2012 | yes | yes | can't tell | yes | no | yes | yes | can't tell | yes | limited |
| Lester 2013 | yes | yes | can't tell | no | no | can't tell | yes | no | yes | valuable |
| Maisey 2008 | yes | yes | can't tell | no | can't tell | yes | no | yes | yes | limited |
| McDonald 2009 | yes | yes | can't tell | no | can't tell | can't tell | yes | no | no | valuable |
| Norman 2014 | yes | yes | can't tell | yes | can't tell | can't tell | yes | no | yes | limited |

| Reviews  (CASP method) | scores on individual items | | | | | | | | | |
| --- | --- | --- | --- | --- | --- | --- | --- | --- | --- | --- |
|  | **1** | **2** | **3** | **4** | **5** | **6** | **7** | **8** | **9** | **10** |
|  |  |  |  |  |  |  |  |  |  |  |
| Gillam 2012 | no | no | yes | yes | can't tell | clearly described | can't tell | yes | yes | no |
| Mehrotra 2009 | yes | can't tell | yes | yes | no | clearly described | can't tell | can't tell | yes | no |

**5. List of 29 identified OBPMs**

| List of identified OBPMs | |  |
| --- | --- | --- |
| *Model name* | ***Country*** | ***Effect study available?*** |
| Alternative Quality Contract (AQC) | USA | yes |
| Bridges to Excellence | USA | no |
| Chipra | USA | no |
| Commissioning for Quality and Innovation (CQUIN) | UK | yes |
| Geisinger Health System | USA | no |
| General Practitioners Consortiums | UK | no |
| Georgia Blues | USA | no |
| Health eHearts | USA | no |
| Home Health P4P | USA | no |
| Hospital Acquired Conditions Reduction Program (HACRP) | USA | no |
| Hospital Quality Incentive Demonstration (HQID) | USA | yes |
| Hospital Readmissions Reduction Plan (HRRP) | USA | yes |
| Hudson Health Plan | USA | yes |
| Long Island Health Network P4P | USA | no |
| Maryland Hospital Acquired Conditions Program | USA | yes |
| Medicare shared savings program (MSSP) | USA | yes |
| Minnesota nursing home P4P | USA | no |
| New Hampshire Accountable Care Organisations | USA | no |
| Norway P4P model | Norway | no |
| Oregon Salem | USA | no |
| Palo Alto Medical Clinic P4P (PAMC P4P) | USA | yes |
| Pharmacist P4P | USA | no |
| Pioneer accountable care organisations (Pioneer ACO) | USA | yes |
| PROMETHEUS payments | USA | no |
| Quality Incentive Program end-stage renal disease | USA | no |
| Quality and Outcome Framework | UK | yes |
| Value Incentive Program (VIP) | Korea | yes |
| Value-Based Purchasing | USA | yes |
| Wellmark IVF P4P | USA | no |
